# 

**Published:** 1883-03

**Authors:** 


### *Death of Dr. Wm. H. Allen.*

At a meeting of the First District Dental Society of the State of New York, held November 7th, 1882, on motion of Dr. Frank M. Odell, a committee of three was appointed to draft suitable resolutions in relation to the death of William H. Allen.

In pursuance of this purpose we present the following:

WHEREAS, in the dispensation of a wise Providence, we are called upon to mourn the loss of one of our most estimable members, it is therefore

*Resolved*, That in the demise of our beloved brother, William H. Allen, we have sustained an irreparable loss; and that we deeply sympathize in this visitation, with his widow and relations.

And further, that a memorial page be set apart in the book of records of this Society, and that copies of the same, and of these resolutions, be sent the journals for publication, and to his family.

FRANK. M. ODELL,
WILLIAM H. ATKINSON, } Committee.
W. T. LA ROCHE.

*University of Maryland, Faculty of Physic,* 76*th Annual Session Commencement,* 1883.—The Commencement Exercises of the Dental Department of the University of Maryland will be held at the Academy of Music, Baltimore City, Thursday March 15th, at 12 M. The Banquet of the University Alumni Association will take place on the same evening. The members of the dental profession are respectfully invited to be present at the Commencement exercises. The large number of graduates of both the Medical and Dental Departments will render the occasion an interesting one.

### To Readers and Correspondents.

Communications intended for publication, and exchange journals and papers, should be addressed to the EDITOR OF THE AMERICAN JOURNAL OF DENTAL SCIENCE, 259 N. Eutaw St., Baltimore, Md. All letters relating to the business of the journal. or containing cash remittances, to be directed to Messrs. SNOWDEN & COWMAN. Articles for publication should be received by the editor at least three weeks previous to the first of the month on which the number in which it is designed for them to appear, is issued.

### MONTHLY SUMMARY.

The Use of Tobacco by Children .... 525
An Idea on Amalgams .... 526
Carbolic Acid Poisoning .... 527
A New Hypnotic and Depresso-Motor .... 528
Influence of Electric Light on Health .... 528

☞ The American Journal of Dental Science will contain fifty pages of reading matter in each number, making a volume of about

*Six Hundred* pages,

EXCLUSIVE OF ADVERTISEMENTS.

THE

### AMERICAN JOURNAL

OF

### DENTAL SCIENCE.

The *Journal* is issued on the first of each month and contains not less than *fifty* pages of reading matter in each number, exclusive of advertisements, making a volume of *six hundred* pages per annum.

In each number will be found Original Communications, Correspondence, Selected Articles, Monthly Summary, Bibliographical Notices and Editorial Matter, and every effort will be exerted to render the *Journal* worthy of the patronage of the Dental profession.

A generous co-operation is respectfully solicited from the members of the profession; and as the *Journal* has now a printing office of its own, which materially lessens the cost of its publication, it will be issued at the reduced subscription price of

**$2.50 Per Annum in Advance.**

Communications are respectfully solicited on all subjects pertaining to the practice of dentistry, as well as reports of cases occurring in dental practice.

### RATES OF ADVERTISING.

| | 1 MO. | 2 MO. | 3 MO. | 6 MO. | 12 MO. |
|---|---|---|---|---|---|
| 1 PAGE. | $15 00 | $22 50 | $30 00 | $50 00 | $100 00 |
| ½ " | 10 00 | 15 00 | 20 00 | 30 00 | 50 00 |
| ¼ " | 7 00 | 11 00 | 15 00 | 20 00 | 30 00 |
| ⅛ " | 5 00 | 8 00 | 11 00 | 15 00 | 20 00 |

All original communications designed for publication, works for review, new instruments for notice, exchanges, &c., should be directed to the editor, 259 N Eutaw St., Baltimore, Md.

All advertisements and subscriptions should be addressed to

SNOWDEN & COWMAN,

*Publishers of the Am. Journal of Dental Science.*

86 W FAYETTE ST. BET. CHARLES & LIBERTY STS.,

BALTIMORE, MD

